# Supplementary solvent irrigation efficacy on filling remnants removal comparing XP-endo Finisher R vs IrriSafe

**DOI:** 10.1038/s41598-021-92175-2

**Published:** 2021-06-16

**Authors:** Inês Ferreira, Pedro S. Babo, Ana Cristina Braga, Maria Ascensão Lopes, Manuela E. Gomes, Irene Pina-Vaz

**Affiliations:** 1 CINTESIS, Faculty of Medicine of University of Porto, Al. Prof. Hernâni Monteiro, 4200-319 Porto, Portugal; 2 3B’s Research Group, I3Bs—Research Institute on Biomaterials, Biodegradables and Biomimetics, University of Minho, Headquarters of the European Institute of Excellence On Tissue Engineering and Regenerative Medicine, AvePark, Parque de Ciência E Tecnologia, Zona Industrial da Gandra, 4805-017 Barco, Guimarães, Portugal; 3 ICVS/3B’s—PT Government Associate Laboratory, 4805-017 Braga/Guimarães, Portugal; 4 Department of Production and Systems, ALGORITMI Center, University of Minho, Campus de Gualtar, 4710-057 Braga, Portugal; 5 REQUIMTE-LAQV, Department of Metallurgical and Materials Engineering, Faculty of Engineering, University of Porto, R. Dr. Roberto Frias, 4200-465 Porto, Portugal; 6 CINTESIS, Faculty of Dental Medicine of University of Porto, Rua Dr. Manuel Pereira da Silva, 4200-393 Porto, Portugal

**Keywords:** Preclinical research, Dental materials, Endodontics, Root canal treatment

## Abstract

This study aimed to compare the efficacy of XP-endo Finisher R and IrriSafe, with a solvent mixture of Methyl ethyl ketone/Tetrachloroethylene (MEK/TCE), in the removal of root filling residues. Twenty-four human mandibular incisors were pair-matched by micro-computed tomography according to volume and aspect ratio. After retreatment, specimens were allocated to two experimental groups (n = 12), according to the supplementary instrument used. The volume of residual filling material after each irrigating step and the time for retreatment was calculated. Statistical analyses were carried out using Mann–Whitney test, with a significance level of 5%. The volume of initial root canal filling material between the groups was similar (p > 0.05). With the final irrigation protocol (NaOCl and EDTA) the volume of the filling remnants decreased significantly (p < 0.05) with no differences between IrriSafe or XP-endo Finisher R (p > 0.05). The additional solvent mixture MEK/TCE increased the efficiency of filling materials reduction, regardless of the agitating instruments employed, IrriSafe or XP-endo Finisher R (p < 0.05). There was no difference between the two groups regarding the time (p = 0.149). Both supplementary instruments were effective in the reduction of filling remnants. The additional step with a solvent mixture of MEK/TCE enabled a total recovery of patency and the achievement of cleaner canals, independently of the agitation instrument.

## Introduction

An increase in the prevalence of apical periodontitis has been recently reported worldwide, in endodontically treated teeth^1^. These findings raise concerns about the quality of restorative and endodontic treatment provided to the populations^2^. To overcome post-treatment disease, non-surgical endodontic retreatment is considered a reliable therapeutic, and is thus the first clinical option^3^. Its main goal is to regain access to the apical foramen by means of a complete removal of the filling materials, to create access and enable a chemo-mechanical debridement of the persisting biofilm, and to reseal it through a new hermetic obturation^4^. Although instrumentation systems have evolved, neither technique is capable of efficiently preparing the complex root canal anatomies^5,6^. Similarly, different contemporary retreatment techniques or enlargement criteria have not been able to render root canals free of potentially infected filling residuals^7^ which might compromise the outcome*.*

A further enlargement of root canal preparation, with particular emphasis in the apical segment, ‘the critical zone of infection’, seems to be unavoidable in view of infection control^8^. However, the balance between the adequate preparation size and its antibacterial benefits or cleanliness improvement, aside with the risk of dentine thickness reduction is still a matter of debate^8–11^. Even though a greater enlargement may lead to a reduction in the unprepared areas^12^, this may not be correlated to a decrease of residual filling materials^13^ or a more predictable cleaning and disinfecting root canals^5,14^. Additionally, retreatment procedures in the apical region remain harder to perform^15–17^.

The introduction of activated irrigating strategies changed the paradigm with reports that smaller apical sizes could result as clean as larger preparations^13,14^. One of the gold-standard agitation techniques is the use of ultrasonic instruments^18^. Despite this, their effectiveness for filling removal shows contradictory results^19–21^. Mechanical agitation driven by novel finishing instruments like XP-endo Finisher R (FKG Dentaire, La Chaux-de-Fonds, Switzerland), specifically developed to boost cleaning during retreatment, were shown to improve the filling removal^15,22–24^. This new variation has a semi-active tip and a larger core diameter (size 30) which distinguishes it from its XP-endo Finisher counterpart. Nevertheless, this possible advantage was not always confirmed^25^. Findings still remain controversial in their comparison with ultrasonic agitation and, in particular, in what concerns the cleaning of the apical area of root canals^22,24^.

Several other factors may influence the persistence of filling materials after retreatment, such as root canals’ anatomy, the retreatment technique, the supplementary finishing procedures or the use of gutta-percha solvents^4,7^. Few studies have assessed the impact of solvents, fearing the messy effect of the softened gutta-percha and the consequently harder and more time-consuming process of cleaning it from root canal walls^26^. However, retreatment files, such as XP-endo Finisher R, are recommended by their manufacturers to be associated to solvents^25^. Other reports about solvents’ benefits in retreatment procedures are inconclusive or controversial^23,27,28^. Ferreira, et al.^13^ showed that a cytocompatible mixture of solvents, Methyl ethyl ketone/Tetrachlorethylene (MEK/TCE), potentiated by sonic agitation, is highly effective for the dissolution of gutta-percha and epoxy resin-based sealer AH Plus, as an additional step for further enhancement of residual filling removal. However, the impact of other agitating techniques, in the amount of filling material remnants, is unknown.

Using microcomputed tomographic (micro-CT) imaging to quantify the filling material volume in the different retreatment stages this study had 3 main objectives: (i) to compare the efficacy of XP-endo Finisher R and IrriSafe with the solvent mixture MEK/TCE, in removing root filling remnants from mandibular incisors’ canals (ii) to assess the additional cleaning effect of a solvent mixture exposure and (iii) to quantify the retreatment time with the use of solvents.

## Results

The Mann–Whitney test confirmed that the obturation volume (root canal filling material) was similar between the groups [Group 1: median (min–max) for 1–5 mm = 0.584 (0.333–0.652) and for 1–10 mm = 2.718 (2.186–3.189); Group 2: median (min–max) for 1–5 mm = 0.502 (0.340–0.599) and for 1–10 mm = 2.438 (2.196–3.982)], which permitted a uniform comparison (p = 0.094 for 1–5 mm and p = 0.325 for 1–10 mm).

**Figure 1:**
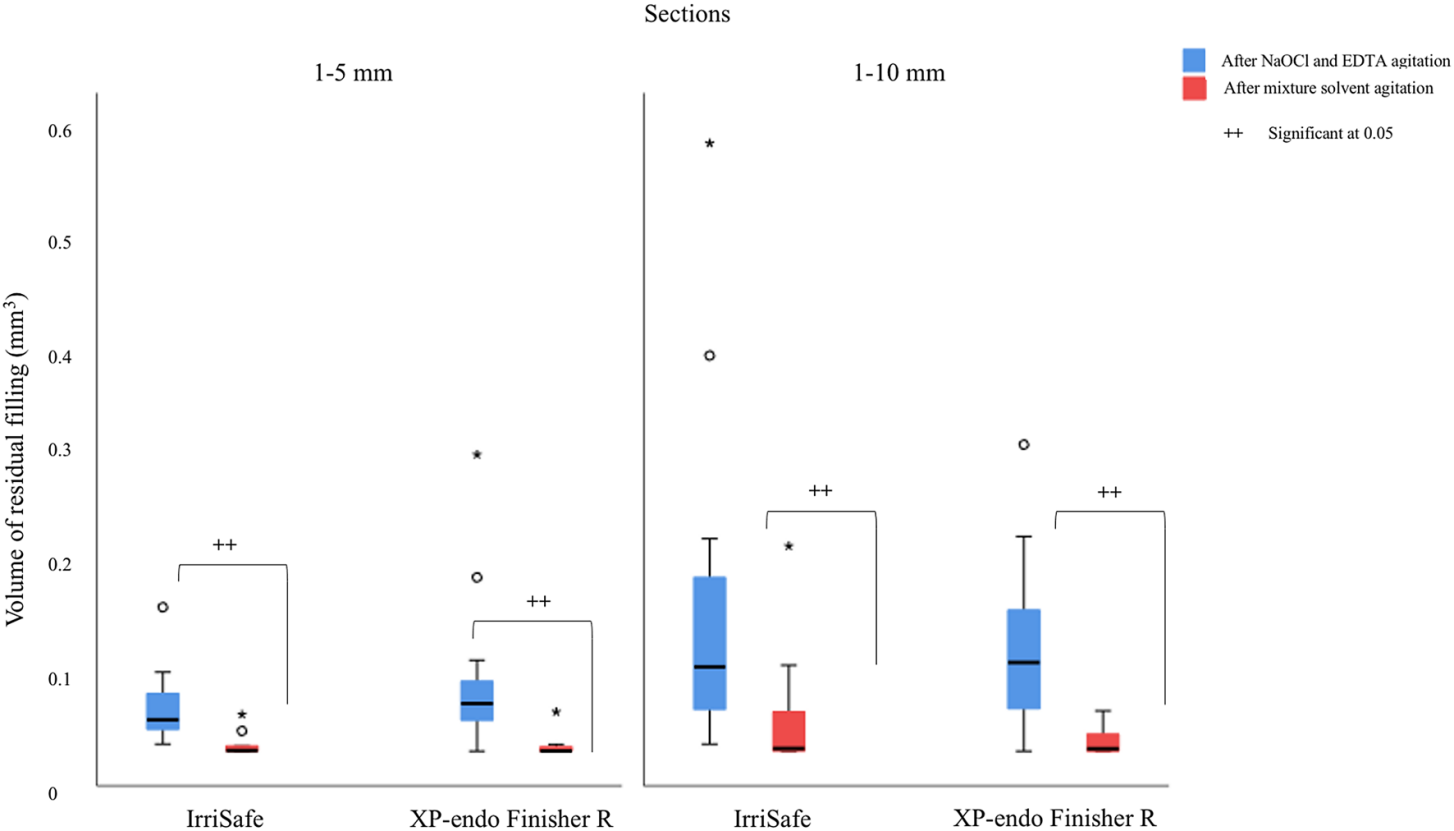
Box plot demonstrating the volume distribution of filling material persisting in the different sections analyzed, according to the type of agitation (IrriSafe or XP-endo Finisher R). (+ + indicate significance at p < 0.05, Mann–Whitney test).

**Figure 2:**
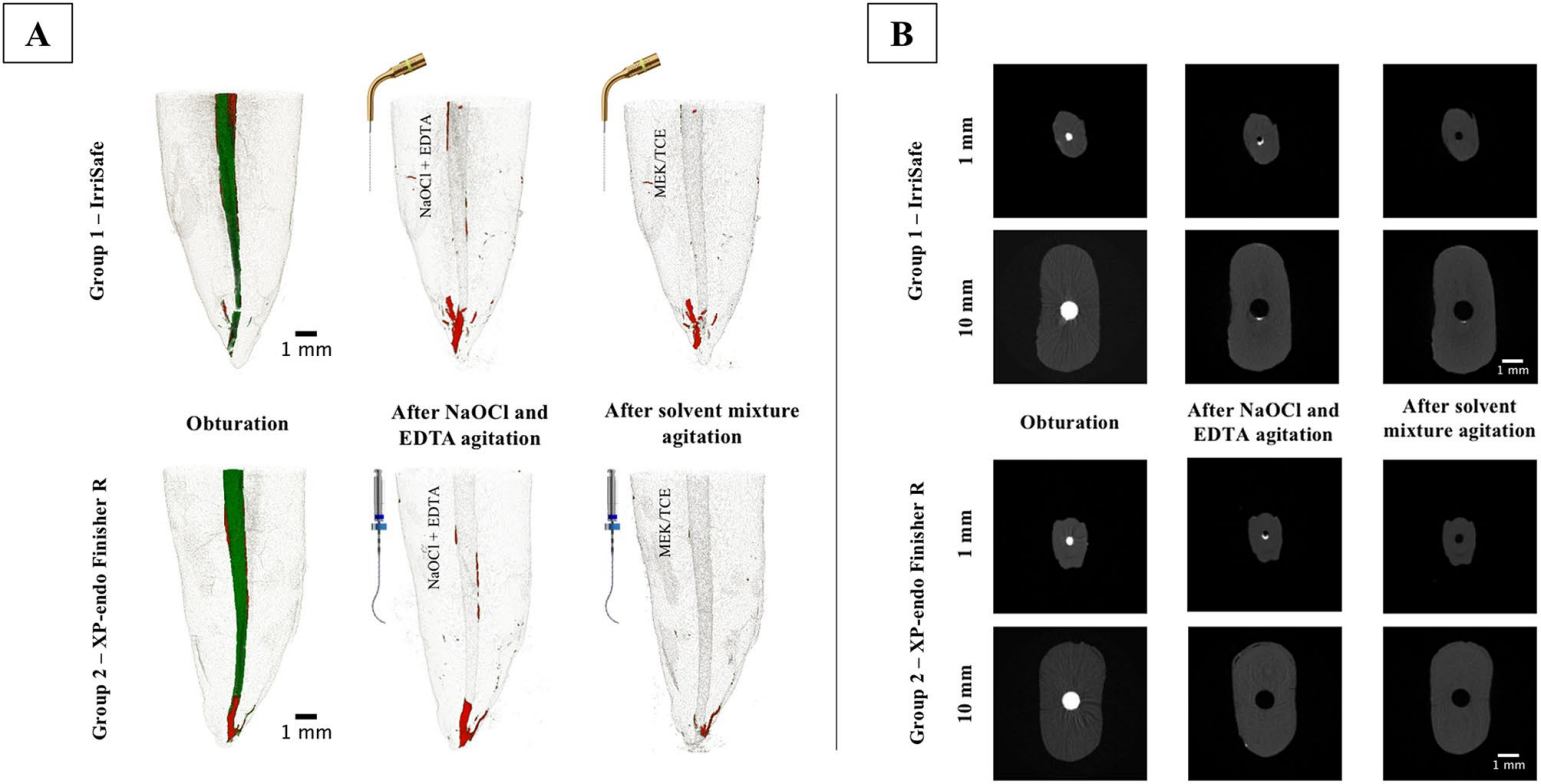
(**A**) Micro-CT reconstructions of the 1–10 mm section of exemplary mandibular incisors specimens subjected to the different procedures. Green represents the gutta-percha and the red the AH Plus sealer. In the Group 1 the irrigants (NaOCl and EDTA) and solvent mixture (MEK/TCE) were agitated by IrriSafe and in the Group 2 by the XP-endo Finisher R. (**B**) Representative cross-sectional micro-CT images of slices at 1 and 10 mm. The 3D reconstructions were made using the software CTVox (version:2.3.0 r810, SkyScan, Bruker, Tuckson, US).

After the re-preparation procedures and the final irrigation protocol with NaOCl and EDTA (agitated by IrriSafe or XP-endo Finisher R) the volume of the filling materials decreased significantly (p < 0.05). There were no differences between IrriSafe or XP-endo Finisher R (p < 0.05), Figs. 1 and 2.

After the supplementary solvent mixture (MEK/TCE) agitation, the volume of filling materials was significantly reduced (p < 0.05), however, the differences were not significant between IrriSafe and XP-endo Finisher R (p > 0.05). There were no differences between both the segments studied, apical of full canal (p > 0.05), Figs. 1 and 2.

The time to complete all retreatment procedures (including re-preparation and application of the solvent) were not significantly different (Table 1).

**Table 1:** Descriptive analysis of the time spent (seconds) to perform all retreatment procedures (including re-preparation and application of the solvent).

	Mean ± SD	Median	Minimum	Maximum	P value
G1 (IrriSafe)	1260.92 ± 23.224	1263.00	1223.00	1300.00	0.149
G2 (XP-endo Finisher R)	1282.50 ± 39.06	1279.50	1235.00	1394.00	

Table 2 shows the filling amount/remnants, in percentage, after the agitation of the conventional final irrigation protocol and after solvent supplementary procedure in the two sections analyzed.

**Table 2:** Amount (%) of filling material removed after the agitation of the conventional final irrigation (NaOCl and EDTA) and after solvent supplementary procedure in the two sections (1–5 mm and 1–10 mm).

Group	Sections	After NaOCl and EDTA agitation				After mixture solvent agitation*			
		Mean ± SD	Median	Minimum	Maximum	Mean ± SD	Median	Minimum	Maximum
XP-endo Finisher R	1–10 mm	96.09 ± 3.23	96.77	88.24	100.00	87.36 ± 19.39	98.44	4.58	100.00
	1–5 mm	86.29 ± 15.32	91.48	45.08	100.00	93.44 ± 13.16	99.58	56.75	100.00
IrriSafe	1–10 mm	94.43 ± 6.73	97.55	79.29	99.74	86.82 ± 17.80	96.33	51.17	100.00
	1–5 mm	92.40 ± 6.16	94.09	75.67	98.90	92.11 ± 13.44	98.19	53.69	100.00
Total	1–10 mm	95.29 ± 5.15	96.86	79.29	100.00	87.08 ± 18.15	98.44	44.58	100.00
	1–5 mm	89.34 ± 11.84	92.62	45.08	100.00	92.75 ± 13.02	98.80	53.69	100.00

*Considering the remaining material after NaOCl and EDTA agitation as a 100%.

## Discussion

The present study evaluated whether the supplementary procedure with a mixture of solvents (MEK/TCE) agitated by XP-endo Finisher R or IrriSafe, improve the removal of residual filling material from mandibular incisors’ root canals. Additionally, the efficacy of the agitating instruments was compared, and the time spent for complete retreatment procedures was also assessed.

The pair-match methodology with micro-tomography introduced higher strict criterion for experimental group formation, minimizing the potential biases presented by the anatomical variations. Likewise, there were no differences in the initial volume or aspect ratio of the root canals selected for both experimental groups. Thus, the volume of filling material (obturation) between groups, assessed by micro-CT scan, was also similar. As in another recent study^22^, two analyses were performed, one for the full canal (considering least 1 mm from the apical foramen to 10 mm short) and the other restricted to the apical segment (considering least 1 mm from the apical foramen to 5 mm short). Filling material remnants were quantified in the different stages of retreatment procedures, through micro-CT scanning, a noninvasive and nondestructive method considered ideal for its evaluation^24^. The present findings revealed a great capacity of chemo-mechanical preparation in removing filling remnants from apical and full canal, with no differences between the agitating instruments tested. Ultrasonic and mechanical activation of NaOCl with XP-endo Finisher R has been thoroughly investigated, being associated with a positive impact in the cleanliness of root canals^20,22–24,29^, even though this might not always be accompanied by an improvement in disinfection^6,30^. The supplementary cleaning capacity of XP-endo Finisher R and ultrasonic agitation of NaOCl and EDTA was also confirmed in the present findings. However, it was still improved by the exposure of a specific mixture of solvents, previously reported^13^. IrriSafe and XP-endo Finisher R, with MEK/TCE, presented a similar efficacy, independently of the analyzed portion the root canal. Other authors corroborated these findings, reporting no differences for filling remnants removal between each anatomical region assessed, through passive ultrasonic agitation technique^24^. Although exploring different sealers, the selection of monorradicular teeth with straight canals and the single-cone filling technique may have influenced these results^19^.

Contrarily, other authors report different performances of the instruments according to the locations of the root canal^20,22^. The explanation given for the similar outcome, restricted to the apical segment, was the tendency presented by root canals to be more circular as they approach the apex^22^. In the present study, the format of round canals could aid to explain the lack of differences between the two segments assessed (Fig. 2B). The type of ultrasonic tip (IrriSafe with an 20/0.1 taper), the power setting (30 kHz), the previous apical size enlargement (0.30 mm (X3—PTN) or the association of the solvent mixture (MEK/TCE) might further explain other discrepancies between the performance of the supplementary instruments^15,22,24^. In order to standardize variables, the volumes of irrigant were similar in both groups.

A reduction of approximately 95.29% for the full canal length and 89.34% for the apical 5 mm, of the initial volume of filling material was verified after chemo-mechanical preparation with NaOCl and EDTA activation either by IrriSafe or XP-endo Finisher R. With the additional solvent agitation, there was an extra filling removal of 87.08% and 92.75%, respectively (Table 2). Additionally, the patency was recovered from 25% of the canals, after NaOCl and EDTA agitation, to 100% after solvent. Although round canals are perceived as retaining a significantly smaller percentage of residual tissue compared to oval shaped canals^31^*,* these percentages are significant, when the fact that it occurred both in full and apical canal areas is taken into account^22^.

The results here presented should be justified by the specificity of the solvents in the mixture, MEK/TCE (1:1), for both epoxy-resin sealer and gutta-percha materials, respectively, shown in previous investigations^13,32–34^. The improved dissolution efficacy obtained was enhanced by agitation, increasing the dissolution profile of the solvents and promoting a better solvent’s dispersion to reach areas not usually touched by endodontic instruments, and thus improving patency and its potential for filling’s removal.

The effect of the time of retreatment has been an argument against the use of solvents, even though it has been reported to be a quicker procedure to reach the working length in its presence^27^. However, the type of solvent or its moment of use were different from the strategy here suggested. According to the present study, despite the use of solvents, the process of retreatment did not take longer than similar studies, regardless of the agitating instrument^19,35^, Table 1. Filling materials may remain in difficult-to-reach areas hampering the disinfection and thus reducing antisepsis and consequently the rate of successful endodontic (re)treatment^36,37^. Preliminary results seem to address antimicrobial/antibiofilm properties of the solvent mixture tested to some of the microorganisms resistant to conventional endodontic disinfecting procedures. Such complementary/antimicrobial properties of the solvent suggested should be deeply investigated, in order to raise retreatment’s success, even without a root canal completely free of residues.

Herein, there was a novel insight with a different rationale of solvents’ application in Endodontics. Previous work was crucial in identifying the dissolution ability of the solvent mixture MEK/TCE, with an improved performance due to the agitation and synergy between the solvents, which seems to also reduce the cytotoxicity of the mixture^32^.

## Conclusions

The positive impact these particular solvents have shown, improving root canal cleanliness, led to move on searching for the best instruments to further improve their performance in clinics. To our knowledge, there are no *ex-vivo* reports about the effect of XP-endo Finisher R or ultrasonics’ performance on solvents’ mixtures in retreatment procedures. Their benefit was shown on the augmented filling removal and regain of patency, independent of the agitating instrument. If the goal of endodontic retreatment is to remove as much as possible filling materials, in order to obtain a fully microbial eradication, development of a novel solvent proposal could be part of the disinfection strategy. Future studies should focus on its efficacy in complex anatomies or with different filling techniques and explore their antibiofilm activity reinforcing all the potential they can bring to improve retreatment’s outcome.

## Methods

All methods were performed in accordance with the relevant guidelines and regulations.

### Specimen selection

**Figure 3:**
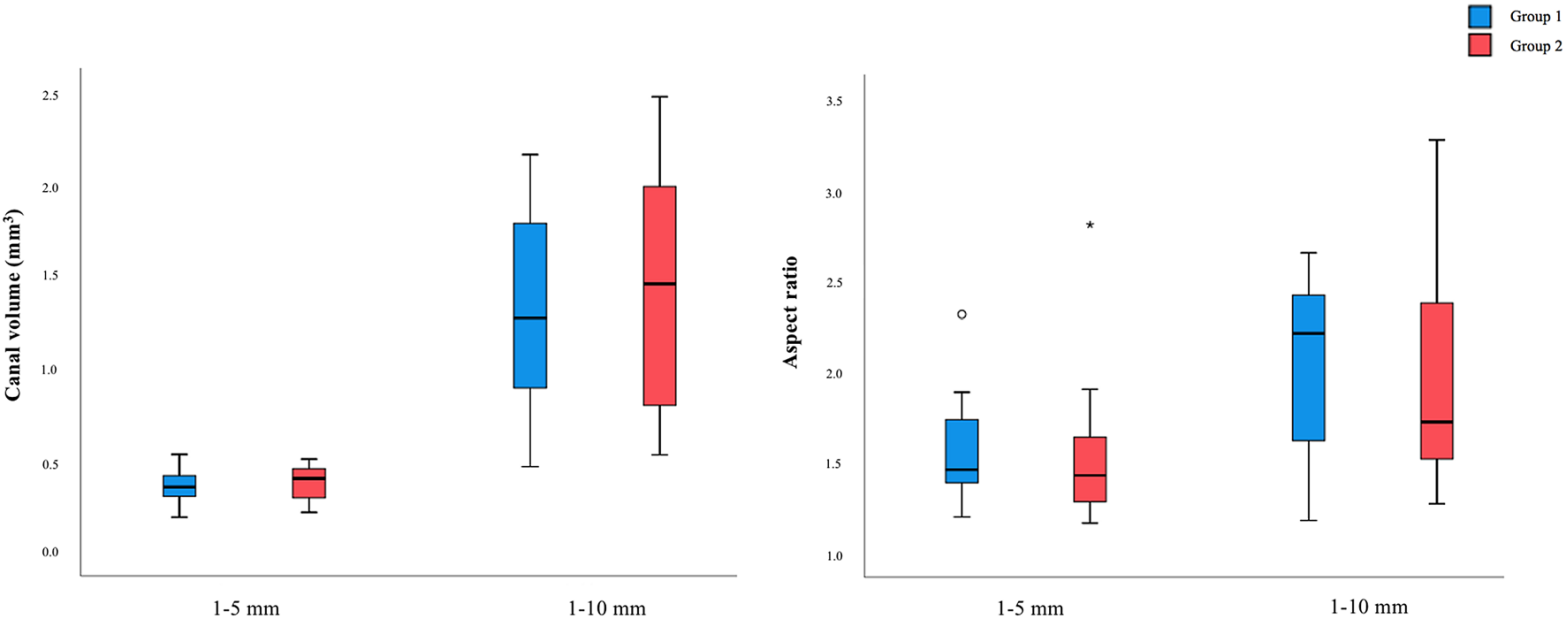
Canal volume (mm^3^) and aspect ratio of the specimens from groups 1 and 2, in both analyzed sections (1–5 mm and 1–10 mm).

After Ethics Committee for Health of the Faculty of Dental Medicine of the University of Porto approval (nº3/2018), twenty-four noncarious, straight similar single-rooted human mandibular incisors, without previously treated canal, root fractures, calcifications, internal or external root resorptions and with mature apex were selected. Mesio-distal and bucco-lingual radiographs were taken to confirm these inclusion criterions. Initial micro-CT scans were done to obtain an overview of the teeth anatomy. In order to create homogeneous groups, the teeth were pair-matched according to their root canal volume and aspect ratio forming two groups of 12. The volume and the aspect ratio between the two groups, considering two sections. (1–5 mm and 1–10 mm), were similar and without statistically significant differences (1–5 mm: for volume p = 0.443 and aspect ratio p = 0.419; 1–10 mm: for volume p = 0.686 and aspect ratio p = 0.644), Fig. 3.

### Root canal preparation and filling

The working length (WL) was determined using a size 10 K-file, which was near 1 mm shorter than the full root-canal length. The roots were shaped with ProTaper Next (X1 and X2 files; Dentsply-Maillefer, Ballaigues, Switzerland) and repetitively irrigated using 3% NaOCl (30G needle—Max-I-Probe, Dentsply International, Inc) between files, with a total of 5 mL of 3% NaOCl. The patency was verified with a size 10 K-type file. The final rinse was done with 2 mL of 3% NaOCl followed by 2 mL of 17% EDTA for 1 min, and, lastly, 1 mL of distilled water. After drying with paper points, the roots were filled with ProTaper Next X2 gutta-percha cone (Dentsply Sirona, Ballaigues, Switzerland) and AH Plus Jet (Dentsply DeTrey, Germany), through the single-cone technique. A lentulo spiral filler was used for laying the sealer. A buccolingual and a mesiodistal radiographies were done in all specimens, to guarantee the consistency of the root filling. A temporary restorative material (IRM; Dentsply Sirona, Ballaigues, Switzerland) was used to seal the access cavities. The samples were kept in 100% humidity at 37 °C for 2 weeks.

### Retreatment and re-preparation procedures

ProTaper Universal Retreatment (D1, D2, and D3; Dentsply Sirona; Ballaigues, Switzerland), following the manufacturers recommendations, were used for the removal of the majority of the obturation. The files were removed from the canal, cleaned with a sponge, and placed again until the required length was reached. Hedström files (Dentsply Sirona, Ballaigues, Switzerland) of size 25 and 30 were used with brushing or circumferential quarter-turn push–pull filling motions, for the refinement of the residual filling material removal, or whenever necessary to reach the WL, which allow a better control of the cleanliness of canal walls. A total of 15 mL of 3% NaOCl was applied. Next, the roots were re-prepared until ProTaper Next X3 file, using 5 mL of 3% NaOCl between files. When no remaining filling materials were detected inside the root canal, on the instruments or floating in the irrigation, the filling materials were considered to be totally removed.

### Supplementary procedures

In Group 1, the IrriSafe (size 20, 0.01 taper; Acteon Group) was used with high power setting equivalent to 30 kHz, according to the manufacturer’s instructions and positioned 1 mm from the working length with an up-and-down motion. In Group 2 the XP-endo Finisher R (size 30, non-tapered; FKG Dentaire) was used up to the WL, in slow up-and-down 7 mm to 8 mm long movements, with 800 rpm and 1 N.cm, pressed against the canal walls.

During this stage it was used 2 mL of 3% NaOCl, followed by 2 mL of 17% EDTA, both agitated for 1 min (with IrriSafe in group 1 or XP-endo Finisher R in group 2). The apical patency was checked in all canals.

Then, all roots were submitted to an additional procedure, using a binary mixture of solvents—MEK/TCE (1:1) (VWR International SAS, France), agitated by IrriSafe (group 1) or XP-endo Finisher R (group 2) for 5 min, for cycles of 1 min. Each root was rinsed with a total of 1 mL of the solvent mixture. The solvent mixture was refreshed after each minute. Finally, all the roots were irrigated with 5 mL of distilled water. At the end of this step, the apical patency was checked again.

All roots were prepared by a single operator, according to the state of the art. All procedures were performed at 37 °C inside a cabinet with a heater that assured temperature control. Each file was used in three roots and then rejected. Another operator who was blinded to the data, performed all micro-CT analysis and a different operator did the statistical analysis.

### Retreatment time

The time required for the retreatment procedure was recorded using a digital chronometer in seconds. It was recorded, including: total active instrumentation, change and cleaning of the instruments and the time devoted to all irrigation process, including supplementary irrigation and solvent procedures.

### Micro-CT imaging analysis

The samples were scanned at four moments: initial micro-CT was performed to obtain an overview of the root canal anatomy, then after obturation, after the conventional final irrigation and following solvent supplementary procedure, using a high-resolution micro-CT scan (Skyscan 1272, Skyscan n.v., Belgium). Series of two-dimensional projections with an isotropic resolution of 10 µm per voxel, were acquired by irradiation with penetrative X-rays, using a voltage of 70 kV and a current of 142 µA, and a 0.5-mm-thick aluminum filter, for an exposure time of 3511 ms, over a rotation range of 360° with a rotation step of 0.45°. The images were reconstructed with the software NRecon (version 1.6.6.0, Bruker, Tuckson, US), using a beam hardening correction of 54% and ring artifact correction as needed. Data was analyzed on the CT analyzer (version 1.4, Skyscan) software, by auto-interpolation of the manually defined regions of interest (ROI), defined as a circle centered in the root canal, to obtained volumes of interest (VOI) corresponding to the apex regions of 1–5 mm and 1–10 mm, which were the essential basis for the quantitative analyses. The three-dimensional models were generated using the software CTVox (version:2.3.0 r810, SkyScan, Bruker, Tuckson, US).

### Statistical analysis

The sample size calculation was done using the G*Power software, (version 3.1.9.6; Franz Faul, Universität Düsseldorf, Düsseldorf, Germany). A previous retreatment study^21^ using single-rooted teeth, was taken as reference to determine the effect size for the present study (i.e., 1.80). An alpha-type error of 0.05, a beta power of 0.95, and a ratio of N2/N1 = 1 were predetermined. Twelve specimens per group were specified as the ideal size.

The statistical analysis was realized using the IBM SPSS Statistic 27.0. software (SPSS Inc, Chicago, Illinois, EUA). The level of significance was set at 5% (p < 0.05). The Kolmogorov–Smirnov test was performed to test for data normality, which determined the use of Mann–Whitney test to compare the initial volume and aspect ratio between the two groups, and also to compare the volume of filling material remnants after NaOCl and EDTA agitation, and after the supplementary use of solvent. The time spent for the retreatment procedures including supplementary procedure were also compared using Mann–Whitney test.
